# The diagnostic accuracy of dopamine transporter SPECT imaging to detect nigrostriatal cell loss in patients with Parkinson’s disease or clinically uncertain parkinsonism: a systematic review

**DOI:** 10.1186/s13550-015-0087-1

**Published:** 2015-03-17

**Authors:** Sven R Suwijn, Caroline JM van Boheemen, Rob J de Haan, Gerrit Tissingh, Jan Booij, Rob MA de Bie

**Affiliations:** Department of Neurology, Academic Medical Center, University of Amsterdam, Meibergdreef 9, PO Box 22660, 1100 DD Amsterdam, The Netherlands; Department of Neurology, Medical Center Haaglanden, Lijnbaan 32, PO Box 432, 2501 CK The Hague, The Netherlands; Department of Clinical Research Unit, Academic Medical Center, University of Amsterdam, Meibergdreef 9, PO Box 22660, 1100 DD Amsterdam, The Netherlands; Department of Neurology, Atrium Medical Center Parkstad, Henri Dunantstraat 5, PO Box 4446, 6401 CX Heerlen, The Netherlands; Department of Nuclear Medicine, Academic Medical Center, University of Amsterdam, Meibergdreef 9, PO Box 22660, 1100 DD Amsterdam, The Netherlands

**Keywords:** Diagnostic accuracy, Clinical diagnosis, Parkinson’s disease, Parkinsonism, Dopamine transporter, Single-photon emission computed tomography, Nigrostriatal cell loss

## Abstract

In specialized movement disorder centers, Parkinson’s disease (PD) is wrongly diagnosed in 6 to 25% of cases. To improve the accuracy of the clinical diagnosis, it is necessary to have a reliable and practical reference standard. Dopamine transporter single-photon emission computed tomography (DAT SPECT) imaging might have the potential (high diagnostic accuracy and practical to use) to act as reference standard in detecting nigrostriatal cell loss in patients with (early stage) parkinsonism. We performed a systematic review to evaluate if DAT SPECT imaging can be used as such. Relevant studies were searched in the MEDLINE and EMBASE databases. Studies were selected when they met the following criteria: (1) all patients were adults with a clinical diagnosis of PD or clinically uncertain parkinsonism and (2) the study reported original data. In addition, studies needed to fulfill one of the two following criteria: (1) patients underwent at least one DAT SPECT and had a neuropathological confirmed diagnosis and (2) patients underwent at least two DAT SPECT scans, performed at least 2 years apart. The search identified 1,649 articles. Eight studies fulfilled our selection criteria and were included in this review. There was only one study including patients with diagnostic uncertainty. Sensitivity and specificity of DAT SPECT imaging to detect nigrostriatal cell loss were 98%. The other studies included patients with a diagnosis of PD in whom there was no uncertainty. In these studies, sensitivity was 100%. Our systematic review indicates that DAT SPECT imaging seems to be accurate to detect nigrostriatal cell loss in patients with parkinsonism.

**Electronic supplementary material:**

The online version of this article (doi:10.1186/s13550-015-0087-1) contains supplementary material, which is available to authorized users.

## Review

### Introduction

Parkinsonism is a clinical syndrome characterized by bradykinesia and at least one of the following symptoms: rest tremor, muscular rigidity, and postural instability. Parkinsonism is most commonly caused by idiopathic Parkinson’s disease (PD) [1,2]. However, parkinsonism is also a prominent feature in, for example, progressive supranuclear palsy (PSP), multiple system atrophy (MSA), psychogenic parkinsonism, dementia with Lewy bodies (DLB), vascular parkinsonism, and drug-induced parkinsonism. There is no definite test to confirm the cause of parkinsonism in clinical practice, except for the vascular causes of parkinsonism. Therefore, diagnostic criteria have been developed in the past 20 years [3-6]. Although the diagnosis is straightforward when patients have a classic presentation, establishing the cause of parkinsonism can be challenging, especially in early stages [7,8]. In specialized movement disorder centers, PD is wrongly diagnosed in 6 to 25% of cases [4,9-11]. General neurologists may even make a misdiagnosis up to 35% [7]. In a community-based study in Wales, only 53% of patients, treated with antiparkinson medication in primary care, met the Queen Square Brain Bank criteria for the clinical diagnosis of PD when reexamined by an experienced movement disorder specialist [12].

The different causes of parkinsonism can be classified into two distinct groups; diseases with nigrostriatal cell loss (e.g., PD, MSA, PSP, and DLB) and diseases without nigrostriatal cell loss (e.g., psychogenic parkinsonism, dystonic tremor, dopa-responsive dystonia, and drug-induced parkinsonism). This classification is of clinical importance since the prognosis is considerably worse in parkinsonism characterized by loss of nigrostriatal cells [13]. Patients without nigrostriatal cell loss, except for dopa-responsive dystonia, do not benefit from treatment with dopaminomimetics and require different treatment [5,13,14].

The current reference standard (or gold standard) of parkinsonism is pathological evaluation. Although accurate, it is not a practical standard for the validation of new diagnostic tools because the time between diagnosis and death is often decades. Therefore, there is a need for an alternative *in vivo* reference standard to detect nigrostriatal dopaminergic cell loss in patients with parkinsonism.

Several different diagnostic tools have been evaluated in their ability to detect nigrostriatal cell loss. The most widely used tests are dopamine transporter single-photon emission computed tomography (DAT SPECT), [^18^ F]DOPA positron emission tomography (PET), and transcranial sonography (TCS).

### Tests used in establishing the cause of Parkinsonism

PET is a radiotracer-based method that can assess the *in vivo* function of the dopaminergic and other neurotransmitter systems. [^18^ F]DOPA PET is a reliable tool to establish nigrostriatal cell loss but not very practical in clinical practice because the technique is only available in a limited number of centers, is expensive, and expertise in cerebral PET imaging is essential [15,16].

DAT SPECT imaging - a practical, less expensive, and more widely available technique than [^18^ F]DOPA PET - has been incorporated in most centers as diagnostic tool [17,18]. DAT tracers like [^123^I]FP-CIT are typically used. Recent studies have shown that DAT SPECT imaging might be a sensitive method to establish nigrostriatal dopaminergic degeneration [19-24]. For example, people with subclinical and even preclinical PD already have clear deficits of the nigrostriatal pathway [21,25-28]. Also, if the scan does not show a nigrostriatal deficit, it is highly unlikely that the patient suffers from symptoms caused by nigrostriatal cell loss [11,29,30]. SPECT with tracers labeling postsynaptic dopamine D_2/3_ receptors (e.g., [^123^I]Iodobenzamide or [^123^I]Iodobenzofuran) shows a relatively low diagnostic accuracy, 59 to 80% sensitivity and 46 to 50% specificity in differentiating PD from non-neurodegenerative diseases [31].

Hyperechogenicity in the area of the midbrain has been consistently found in 79 to 90% of patients with PD using TCS [27,32-36]. However, in 6 to 12% of healthy volunteers, hyperechogenicity of the substantia nigra is also found [33,36,37]. This is even higher (16%) in patients with essential tremor [38].

To summarize, DAT SPECT imaging appears to be the most suitable candidate to act as reference standard in detecting nigrostriatal cell loss in patients with (early stage) parkinsonism. Therefore, we performed a systematic review to assess the diagnostic accuracy of DAT SPECT imaging.

### Methods

#### Literature search

We searched the electronic databases MEDLINE and EMBASE using the entire time scale up to July 2013. Both search strategies are included in Additional file 1. Furthermore, we searched the reference lists of all relevant articles to identify additional published studies for possible inclusion in the review. We did not impose any language restrictions.

#### Selection of studies

The list of titles and abstracts was screened by two independent reviewers (SRS and CVB) for eligible studies.

Studies were selected according to the following inclusion criteria: (1) all patients were adults with a clinical diagnosis of PD or clinically uncertain parkinsonism and (2) the study reported original data. In addition, studies needed to fulfill one of the two following criteria: (1) patients underwent at least one DAT SPECT during their life and had postmortem neuropathological evaluation or (2) patients underwent at least two DAT SPECT scans, performed at least 2 years apart. We anticipated that there were only a few studies with DAT SPECT imaging and neuropathological evaluation as reference test (gold standard) available. Therefore, we also included studies that used a second DAT SPECT scan.

The interval of 2 years was chosen to make sure the second DAT SPECT scan would detect any nigrostriatal cell loss. With an annual decline of 5.5 to 7.1% of dopaminergic neurons in PD and even more in atypical parkinsonian syndromes, a scan at least 2 years later would show a marked decline if nigrostriatal cell loss was present but not visible on the baseline scan [23,39].

All study designs were included with the exception of case reports, case series (less than five patients), and case-control studies. We did not impose restrictions regarding the type of radiotracer used (e.g., [^123^I]FP-CIT or [^123^I]β-CIT). If investigators published several reports based on data from a single study population, we selected the most complete report. Articles were excluded if information on diagnostic accuracy (e.g., sensitivity and specificity) could not be derived from the data and could not be obtained from the authors. In all cases, disagreements about study selection were resolved by consensus and a third reviewer (JB) was consulted if disagreement persisted.

#### Data extraction and risk of bias

Data were extracted by the two reviewers (SRS and CVB) independently using an extraction form designed for this review. We extracted data on the diagnostic accuracy of the studies, the baseline characteristics of studied patients (e.g., disease duration, age at imaging, Hoehn and Yahr score, and Unified Parkinson’s Disease Rating Scale score). We also extracted data on the SPECT technique, which radiotracer was used, handling of DAT interfering medication, patient preparation, test interpretation, technical failures, and assessors (e.g., knowledge of other test results). Results were compared, and discrepancies between the two reviewers were resolved in a meeting.

The Quality Assessment of Diagnostic Accuracy Studies 2 (QUADAS 2) checklist was used to assess the methodological quality of the included studies. The QUADAS 2 tool is structured in a series of questions which should be answered ‘yes’, ‘no’, or ‘unclear’ and aims to evaluate the risk of different types of bias [40].

#### Statistical analysis and data synthesis

For each study, patient and study characteristics were summarized using descriptive statistics. Diagnostic accuracy was described in terms of sensitivity and specificity rates with their 95% confidence intervals (CI). We did not perform a meta-analysis because we included a heterogeneous group of patients with a clinical diagnosis of PD as well as with clinically uncertain parkinsonism in this review. This alters the spectrum of disease and non-disease in the population, which may have strong impact on test accuracy.

### Results

#### Literature search and study selection

**Figure 1:**
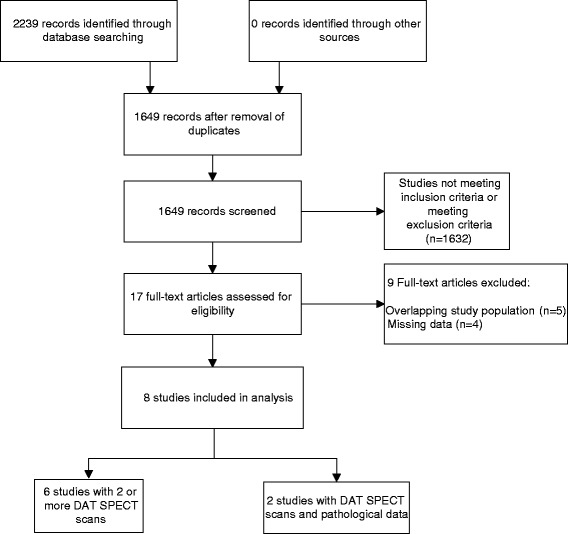
**Flowchart of eligible studies.**

Figure 1 shows the results of the Additional file 1 search and the study selection. We identified 2,239 articles. After duplicate removal, 1,649 articles remained. A total of 1,632 articles were excluded because they did not fulfill our selection criteria. The large majority of these studies were excluded because they did not employ multiple DAT SPECT scans or neuropathological evaluation. Seventeen articles were selected for further review. Of these, five were excluded because the study population overlapped with more complete or more recent publications. We contacted the authors of five additional studies that had missing data [41-44]. One author responded [23]. The other four articles were excluded. In the end, eight studies fulfilled our selection criteria.

#### Study characteristics

The characteristics of the included studies are shown in Table 1. The eight included studies involved a total of 235 patients. There was only one study that assessed patients with clinically uncertain parkinsonism [45], all other studies included patients with a clinical diagnosis of PD (five studies) or pathologically proven PD (two studies). The mean disease duration at first SPECT imaging ranged from 2.4 to 7.9 years.

**Table 1:** **Patient and study characteristics**

**Author, year**	**Number of patients**	**Target population**	**Inclusion criteria**	**Prospective or retrospective?**	**Reference standard**	**Mean disease duration at 1st scan (y ± SD)**	**Mean age at imaging (y ± SD)**	**Mean Hoehn and Yahr score**	**Mean UPDRS motor score at imaging (OFF state, 0 to 108)**	**Mean follow-up between scans (y + SD)**	**Radiotracer**	**SPECT judged visually, template or drawn**	**Drug stopped appropriate before SPECT?**	**SPECT judged blindly for results of reference standard?**
Chouker, 2001 [20]	8	Clinical PD	Step 1 + 2 UKPDS criteria + response to dopaminomimetics	Prospective	2nd DAT SPECT	3.6^¥^, 1 to 6*	57.0, 40 to 76*	2.0 ± 0.8	-	2.0	IPT	Template	Unclear	Unclear
Marek, 2001 [22]	32	Clinical PD	Step 1 + 2 UKPDS criteria	Prospective	2nd DAT SPECT	2.5^¥^ ± 2.4	60.0 ± 11.7	1.8 ± 0.7	18.2 ± 8.7	2.3 ± 0.9	Beta	Drawn	Unclear	Yes
Pirker, 2002 [23]	36	Clinical PD	Step 1 + 2 UKPDS criteria	Prospective	2nd DAT SPECT	4.7^¥^ ± 2.9	60.0 ± 11.0	2.1 ± 0.4	-	2.2 ± 0.3	Beta	Drawn	Yes	Yes
Marshall, 2009 [49]	99	Clinically uncertain parkinsonism	Step 1 UKPDS criteria	Prospective	2nd DAT SPECT	2.4^ǂ^	60.8 ± 4.8	1.5 ± 0.3	9.6 ± 1.3	3.0	fpcit	Visual	Yes	Yes
Isaias, 2010 [48]	13	Clinical PD	Steps 1 to 3 UKPDS criteria	Prospective	2nd DAT SPECT	5.0^¥^ ± 2.8	63.4 ± 8.5	-	17.2 ± 6.0	3.2 ± 1.0	fpcit	Template	Yes	Unclear
Perju-Dumbrava, 2012 [46]	8	Pathologically proven PD	Neuropathological diagnosis of PD + DAT scan during lifetime	Retrospective	Pathological evaluation	4.1^¥^ ± 4.8	68.0 ± 7.2	-	-	3.7 ± 3.0^ʠ^	Beta	Drawn	Unclear	Unclear
Colloby, 2012 [47]	12	Pathologically proven PDD	PDD according to McKeith criteria + DAT scan during lifetime	Retrospective	Pathological evaluation	7.9^¥^ ± 6.3	70.8 ± 4.3	-	35.8 ± 11.9	3.3 ± 1.7^ʠ^	fpcit	Template	Unclear	Unclear
Eggers, 2012 [45]	27	Clinical PD	Steps 1 to 3 UKPDS criteria	Retrospective	2nd DAT SPECT	3.9^¥^	61.7 ± 11.2	-	25.9 ± 5.2	2.5 ± 0.7	fpcit	Template	Yes	Yes

PD, Parkinson’s Disease; SD, standard deviation; UPDRS, Unified Parkinson’s disease rating scale; beta, [^123^I] β-carboxymethyoxy-3-beta-(4-iodophenyl) tropane; fpcit, [^123^I]-fluoropropyl-carbomethoxy-3β-4-iodophenyltropane; IPT, ^123^I-N-(3-iodopropen-2-yl)-2-carbomethoxy-3β-(4-chlorophenyl) tropane; SPECT, single-photon emission computed tomography; CI, confidence interval; PDD, Parkinson’s disease dementia; DAT, dopamine transporter; UKPDS, United Kingdom Parkinson’s Disease society.

^*^Full range instead of standard deviation.

^¥^Disease duration calculated from diagnosis.

^ǂ^Disease duration calculated from first symptoms.

^ʠ^In this study, the interval is referring to the time between DAT SPECT imaging and pathological evaluation.

Two studies used pathological evaluation as the reference standard [46,47]. The other six studies used a second DAT SPECT scan, at least 2 years later, as surrogate reference standard [20,22,23,45,48,49]. The studies using pathological evaluation were retrospective studies. All but one of the studies that used a second DAT SPECT scan was prospective studies.

#### Risk of bias

The risk of bias in the included studies is shown in Table 2. Most studies had at least one design flaw. Five out of the eight studies had an unclear or a high risk of bias regarding patient selection due to a poor description of patient recruitment. In three out of eight studies, there was a concern regarding patient flow. There was a selection of patients that received the reference standard (second DAT SPECT or pathological evaluation). In two studies, we found previous reports with more patients than the ones selected. However, the reasons for exclusion of the extra patients were not mentioned in the manuscript. In four of the eight studies, it was unclear if the assessor of the index test was blinded for the results of the reference test and/or clinical diagnosis.

**Table 2:** **Risk of bias as assessed with the Quadas-2 tool**

**Study**	**Risk of bias**				**Applicability concerns**		
	**Patient selection**	**Index test**	**Reference standard**	**Flow and timing**	**Patient selection**	**Index test**	**Reference standard**
Chouker, [20]	+	?	?	+	+	+	+
Marek, [22]	?	+	+	−	+	+	+
Pirker, [23]	+	+	+	+	+	+	+
Marshall, [49]	+	+	+	+	+	+	+
Isaias, [48]	?	?	?	−	+	+	+
Perju-Dumbrava, [46]	−	?	?	+	+	+	+
Colloby, [47]	−	?	?	+	+	+	+
Eggers, [50]	−	+	+	−	+	+	+

+Low risk, −High risk, ?Unclear Risk.

#### Diagnostic accuracy

Only in the study of patients with clinically uncertain parkinsonism, a high diagnostic accuracy of DAT SPECT imaging was observed with sensitivity and specificity rates of 98%. Only in two out of 99 patients, DAT SPECT results at follow-up differed compared to the initial DAT SPECT scan [49]. In the other seven studies - that included patients with a clinical or neuropathological diagnosis of PD - DAT SPECT imaging had sensitivity rates of 100%. In six of these seven studies, specificity rates could not be calculated as the reference tests indicated all patients had nigrostriatal cell loss. In the study of Marek and colleagues, 30 patients showed nigrostriatal cell loss at imaging and two did not. Sensitivity and specificity rates were both 100%. An important finding of all seven studies is that the results did not change in the course of 2.0 to 3.7 years.

### Discussion

Our study indicates that DAT SPECT imaging may be accurate in detecting the loss of nigrostriatal dopaminergic cells. As anticipated, there are only two studies having both DAT SPECT imaging and pathological evaluation. None of these studies performed DAT SPECT imaging in early-stage clinical PD or in patients with clinically uncertain parkinsonism. However, these two studies show that DAT SPECT imaging at medium-long disease duration (4.1 to 7.9 years) is able to detect nigrostriatal cell loss [46,47].

A limitation of this review is that the majority of included studies performed serial DAT SPECT imaging in patients with a high suspicion of PD. These studies were designed to measure disease progression. In one of the studies, two patients had a negative reference standard. In the other studies, all included patients had a positive reference standard and consequently specificity rates of the index test could not be calculated. However, these results are inline with the only study that evaluated the diagnostic accuracy in patients with clinically uncertain parkinsonism (sensitivity and specificity 98%).

Another limitation is that in four studies it is not clear whether the investigators assessing the SPECT images were blinded for signs and symptoms or for the reference standard.

Other (systematic) reviews on the accuracy of DAT SPECT imaging showed somewhat lower sensitivity (79 to 100%) and specificity (80 to 100%) [18,50,51]. These reviews used the clinical diagnosis at follow-up as reference standard. The clinical diagnosis is accurate in 65 to 94% of the patients compared to the final pathological diagnosis [4,9-11]. The accuracy of the clinical diagnosis increases with the duration of symptoms [49,52]. However, most studies and clinical trials use the clinical diagnosis 3 to 36 months after the initial diagnosis as reference standard [53-56]. This inaccurate reference standard might explain that the accuracy of DAT SPECT imaging is somewhat lower in these studies.

Therefore, there is a need for an alternative *in vivo* reference standard to detect nigrostriatal dopaminergic cell loss in patients with parkinsonism. To evaluate new screening methods, and ultimately the accuracy of the clinical criteria for (early) PD, a reliable and practical reference standard to detect nigrostriatal cell loss is needed [57-60]. The current reference standard (gold standard), pathological evaluation, is accurate, however, not very practical to use to evaluate the accuracy of the clinical criteria because the time between the clinical diagnosis and pathological diagnosis is typically long.

For clinical trials, it will be better and more practical to have an accurate *in vivo* reference standard to ensure no patients with other diagnoses are included. If disease-modifying therapies become available, it will also be desirable to identify patients as early as possible, maybe even in a premotor phase [61,62]. Especially considering that using the clinical criteria, based on motor symptoms, more than 50% of the dopamine producing neurons are lost [61,62].

## Conclusions

It seems that DAT SPECT imaging could be used as an *in vivo* reference standard to detect nigrostriatal cell loss and evaluate new diagnostic (screening) methods. However, considering only one study included patients with diagnostic uncertainty more diagnostic accuracy studies are needed to confirm this finding.

## Additional file

Additional file 1:
**MEDLINE and EMBASE search strategies.**
