# Supplementary material for: The diagnostic accuracy of dopamine transporter SPECT imaging to detect nigrostriatal cell loss in patients with Parkinson’s disease or clinically uncertain parkinsonism: a systematic review
Source: EJNMMI Res. 2015 Mar 17;5:12. doi: 10.1186/s13550-015-0087-1 (PMC4385258; doi:10.1186/s13550-015-0087-1)
Supplement: Additional file 1: — MEDLINE and EMBASE search strategies. [file 13550_2015_87_MOESM1_ESM.doc]

**MEDLINE search strategy**

| **Line #** | **Terms** |
| --- | --- |
| 1 | (DAT adj SPECT).ti,ab. |
| 2 | (dopamine adj3 SPECT).ti,ab. |
| 3 | "dopamine active transporter".ti,ab. |
| 4 | (DT adj2 SPECT).ti,ab. |
| 5 | (FP-CIT adj3 SPECT).ti,ab. |
| 6 | "single photon emission computed tomography".mp. |
| 7 | dopamine.mp. |
| 8 | 6 and 7 |
| 9 | exp Tomography, Emission-Computed, Single-Photon/ |
| 10 | exp Dopamine/ |
| 11 | 9 and 10 |
| 12 | Beta-CIT SPECT.ti,ab. |
| 13 | 1 or 2 or 3 or 4 or 5 or 8 or 11 or 12 |
| 14 | exp Tomography, Emission-Computed, Single-Photon/ |
| 15 | exp Dopamine/ |
| 16 | exp Dopamine Plasma Membrane Transport Proteins/ |
| 17 | 15 or 16 |
| 18 | 14 and 17 |
| 19 | (123I adj5 SPECT).ti,ab. |
| 20 | animals/ not humans/ |
| 21 | exp Parkinson Disease/ or parkinson*.ti,ab. |
| 22 | exp "Sensitivity and Specificity"/ |
| 23 | specificit*.tw. |
| 24 | accuracy.tw. |
| 25 | false negative.tw. |
| 26 | 22 or 23 or 24 or 25 |
| 27 | pathology.mp or exp pathology/ |
| 28 | (dopamine* adj2 degeneration$).tw. |
| 29 | (post-mortem adj2 diagnosis).tw. |
| 30 | exp autopsy/ |
| 31 | necrops$.tw. |
| 32 | obduction$.tw. |
| 33 | ((post mortem or postmortem) adj2 examination).tw. |
| 34 | (Braak$ adj2 stage$).tw. |
| 35 | 27 or 28 or 29 or 30 or 31 or 32 or 33 or 34 |
| 36 | 13 or 18 or 19 |
| 37 | 21 and 36 |
| 38 | 26 or 35 |
| 39 | 36 and 38 |
| 40 | 37 not 20 |
| 41 | 39 not 20 |
| **42** | **40 and 41**  **EMBASE search strategy**   | **Line #** | **Terms** | | --- | --- | | 1 | (DAT adj SPECT).ti,ab. | | 2 | (dopamine adj3 SPECT).ti,ab. | | 3 | "dopamine active transporter".ti,ab. | | 4 | (DT adj2 SPECT).ti,ab. | | 5 | (FP-CIT adj3 SPECT).ti,ab. | | 6 | "single photon emission computed tomography".mp. | | 7 | dopamine.mp. | | 8 | 6 and 7 | | 9 | exp single photon emission computer tomography/ | | 10 | exp dopamine/ | | 11 | 9 and 10 | | 12 | Beta-CIT SPECT.ti,ab. | | 13 | 1 or 2 or 3 or 4 or 5 or 8 or 11 or 12 | | 14 | exp single photon emission computer tomography/ | | 15 | exp dopamine/ | | 16 | exp dopamine transporter/ | | 17 | 15 or 16 | | 18 | 14 and 17 | | 19 | (123I adj5 SPECT).ti,ab. | | 20 | animal/ not human/ | | 21 | exp Parkinson's Disease/ or parkinson*.ti,ab. | | 22 | exp "sensitivity and specificity"/ | | 23 | specificit*.tw. | | 24 | accuracy.tw. | | 25 | false negative.tw. | | 26 | 22 or 23 or 24 or 25 | | 27 | pathology.mp or exp pathology/ | | 28 | (dopamine* adj2 degeneration$).tw. | | 29 | (post-mortem adj2 diagnosis).tw. | | 30 | exp autopsy/ | | 31 | necrops$.tw. | | 32 | obduction$.tw. | | 33 | ((post mortem or postmortem) adj2 examination).tw. | | 34 | (Braak$ adj2 stage$).tw. | | 35 | 27 or 28 or 29 or 30 or 31 or 32 or 33 or 34 | | 36 | 13 or 18 or 19 | | 37 | 21 and 36 | | 38 | 26 or 35 | | 39 | 36 and 38 | | 40 | 37 not 20 | | 41 | 39 not 20 | |
